# Persistence phenotype of adherent-invasive *Escherichia coli* in response to ciprofloxacin, revealing high-persistence strains

**DOI:** 10.15698/mic2025.07.854

**Published:** 2025-07-11

**Authors:** Valeria Pérez-Villalobos, Roberto Vidal, Marcela A. Hermoso, Paula Bustamante

**Affiliations:** 1 Molecular and Cellular Microbiology Laboratory, Instituto de Ciencias Biomédicas, Facultad de Ciencias de la Salud, Universidad Autónoma de Chile, Chile.; 2 Programa de Microbiología y Micología, Instituto de Ciencias Biomédicas, Facultad de Medicina, Universidad de Chile, Chile.; 3 Laboratory of Innate Immunity, Program of Immunology, Institute of Biomedical Sciences, Faculty of Medicine, Universidad de Chile, Santiago, Chile.; 4 Department of Gastroenterology and Hepatology, University Medical Center Groningen, Groningen, Netherlands.

**Keywords:** antibiotic, persistence, persister cell, AIEC, chronic diseases, Crohn´s disease

## Abstract

Persister cells are a subpopulation of bacteria capable of surviving antibiotic treatments and are thought to contribute to disease chronicity and symptom relapse of chronic conditions. Crohn’s disease (CD) is a multifactorial chronic inflammatory condition of the gastrointestinal tract, and adherent-invasive *Escherichia coli* (AIEC) have emerged as a key contributor to its pathogenesis. AIEC can survive, replicate, and produce persister cells within macrophages; however, beyond the LF82 reference strain, little is known about the persistence phenotype and its variability among AIEC strains. In this study, the survival of two AIEC reference strains was analyzed following ciprofloxacin treatment, a fluoroquinolone antibiotic commonly used in CD therapy. In addition, four AIEC clinical isolates and two non-AIEC *E. coli* pathotypes were included for comparison. We investigated the roles of the resident antibiotic resistance plasmid, the stress response protein HtrA, and macrophage-induced persister formation. Our results revealed broad variability in persister cell formation among AIEC strains. Remarkably, the reference NRG857c strain exhibits a threateningly high-persistence phenotype, with persistence levels 200-fold higher than LF82 and certain clinical isolates. Neither the antibiotic resistance plasmid nor HtrA were required for this phenotype. Moreover, unlike LF82, NRG857c did not exhibit increased persistence following macrophage internalization. Overall, our findings demonstrate the presence of distinct persistence phenotypes among AIEC strains and identify NRG857c as a high-persistence variant. These observations underscore the need to consider bacterial persistence in the management of CD, particularly given the potential presence of AIEC strains with elevated persistence capabilities.

## Abbreviations

AIEC - adherent-invasive Escherichia. coli,

CD - Crohn’s disease,

EPEC - enteropathogenic E. coli,

UPEC - uropathogenic E. coli.

## INTRODUCTION

Recurrence of symptoms in chronic diseases is mainly due to relapse rather than reinfection, as numerous bacterial infections can persist in the host for long periods and are not cleared by antibiotic treatments 1. A subpopulation of transient antibiotic-tolerant bacteria, known as persister cells, is thought to be a key actor in these processes 2. Persister are slow-growing or growth-arrested bacterial cells, with a decreased but still active metabolism 3, whose formation has been linked to the stringent response through (p)ppGpp, the SOS response, and toxin-antitoxin systems 2, without lack of controversy 4. In addition, mutations can increase the level of persistence, as the recognized *hipA7* variant 5 and other high-persistence mutants have been observed in patients subjected to repeated antibiotic treatments 6,7.

The presence of persisters during infections has been observed in adherent-invasive *Escherichia coli* (AIEC) 8, an *E. coli* pathotype with high prevalence in Crohn´s disease (CD) patients 9. Bacterial contribution is key for the onset of CD, promoting chronic inflammatory relapses 10, and consequently, ciprofloxacin and/or metronidazole treatments have shown positive results in clinical trials 11.

Pathogenic mechanisms of AIEC are not fully understood, but it is characterized by its ability to adhere and to invade intestinal epithelial cells, and colonize macrophages 12,13. Genome-wide comparison with reference AIEC strains, LF82 and NRG857c 14, revealed an evolutionary relationship, with strain-specific genetic elements encoded on the chromosome and large extrachromosomal plasmids unique to each isolate. Although AIEC members display genetic variability 15, a main characteristic of the pathotype is the ability to survive and replicate within macrophages 13, where the protease HtrA plays an important role 16. Besides, macrophages induce the formation of LF82 persister cells 8.

Considering the diversity found among AIEC members and that persistence has been studied exclusively in the LF82 strain, this study aimed to analyze the persistence phenotype of AIEC reference strains and clinical isolates, comparing with other *E. coli* pathotypes, and to assess whether chromosomal or extrachromosomal genetic factors were involved, along with the effect of macrophage in formation of AIEC persister cells.

## RESULTS 

### Reference AIEC strains exhibit distinct persistence phenotypes

**Figure 1:**
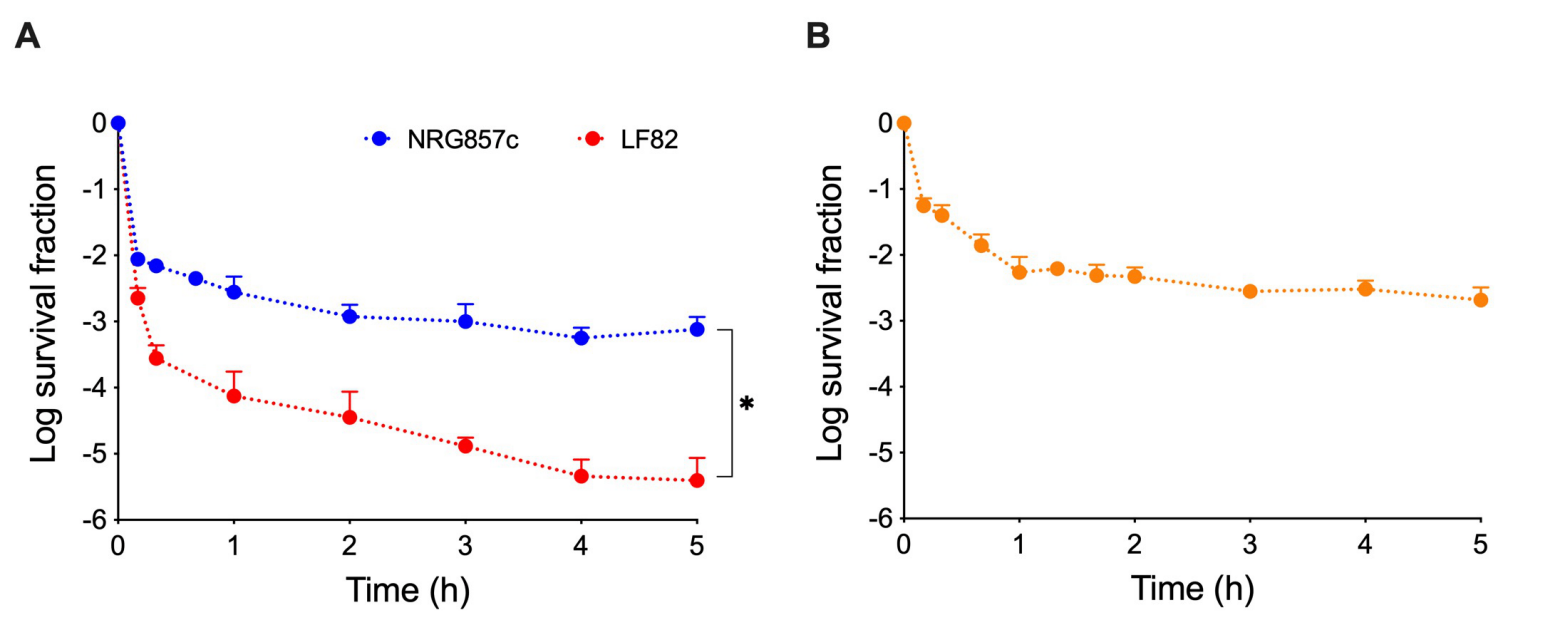
FIGURE 1: Time-killing curves of reference AIEC strains. **(A)** NRG857c and LF82 strains or **(B)** NRG857c(Cu) strain, a derivative of NRG857c cured of its multi-resistance plasmid, were grown in LB broth, challenged with 50-fold MIC of ciprofloxacin, and survival was monitored at indicated times. Data points are mean values of three independent experiments, and standard deviations are represented by error bars above the mean. Student’s t-test was performed for NRG857c and LF82 strains data at 5-hours post-treatment (*P < 0.05).

We analyzed the persistence phenotype of reference AIEC strains, LF82 and NRG857c, in response to ciprofloxacin, a fluoroquinolone antibiotic commonly used in CD treatment 11. We assessed the survival of strains exposed to different antibiotic concentrations and found that the persister plateau was comparable when using either 50- or 100-fold the MIC (Fig. S1). Thereafter, we used an antibiotic concentration equivalent to 50-fold the MIC for all further experiments (**Table 1**), in accordance with serum levels documented in patients following oral administration of ciprofloxacin 17. Although NRG857c and LF82 behaved with the characteristic biphasic killing curve of persister cell formation, their surviving fractions after the ciprofloxacin challenge differed significantly (**Figure 1A**). Remarkably, at 5-hour post-antibiotic challenge, ~0.08% of the NRG857c population survived, 200-fold compared to LF82 at the same time point (**Figure 1A**). Of note, we got similar survival fractions for NRG857c when a MOPS-minimal medium was used 18, even with lower antibiotic concentrations (Figure S2). Surviving NRG857c bacteria did not acquire antibiotic resistance during the experiment (Table S1) and behaved as the original bacterial culture following antibiotic challenge (Figure S3), thus confirming that they correspond to persister cells. Overall, our results show that AIEC reference strains have contrasting persistence phenotypes, with NRG857c having a remarkably high persistence level.

**Table 1:** Ciprofloxacin MIC values of bacterial strains used in this study.

**Strain**	**MIC (µg/mL) ± SD**
LF82	0.0210 ± 0.0080
LF82 Δ*htrA*::Km	0.0234 ± 0.0191
NRG857c	0.0117 ± 0.0043
NRG857c Δ*htrA*::Gm	0.0104 ± 0.0040
NRG857c(Cu)	0.0160 ± 0.0000
CD1a	0.0078 ± 0.0000
CD2a	0.0078 ± 0.0000
CD6b	0.2188 ± 0.0579
CD6r	0.1250 ± 0.0000
E2348/69 (EPEC)	0.2500 ± 0.0000
CFT073 (UPEC)	0.0130 ± 0.0040

### The NRG857c multi-resistance plasmid is dispensable for persistence

NRG857c harbors a multi-resistant plasmid, pO83_CORR 14, and to elucidate if this element was involved in the NRG857c high-persistence phenotype, a NRG857c(Cu) plasmid-cured strain 19 was used. Absence of pO83_CORR did not affect growth (Figure S4) neither ciprofloxacin MIC value (**Table 1**). We found that the plasmid-cured strain did not show significant differences in persistence in comparison to the wild-type strain, with ~0.21% of the bacterial population surviving after 5-hour post-antibiotic treatment (**Figure 1B**). This result demonstrates that the high-persistence phenotype of NRG857c does not rely on the carriage of pO83_CORR, or any gene encoded by it.

### Isolates within the AIEC pathotype display a range of persistence phenotypes

**Figure 2:**
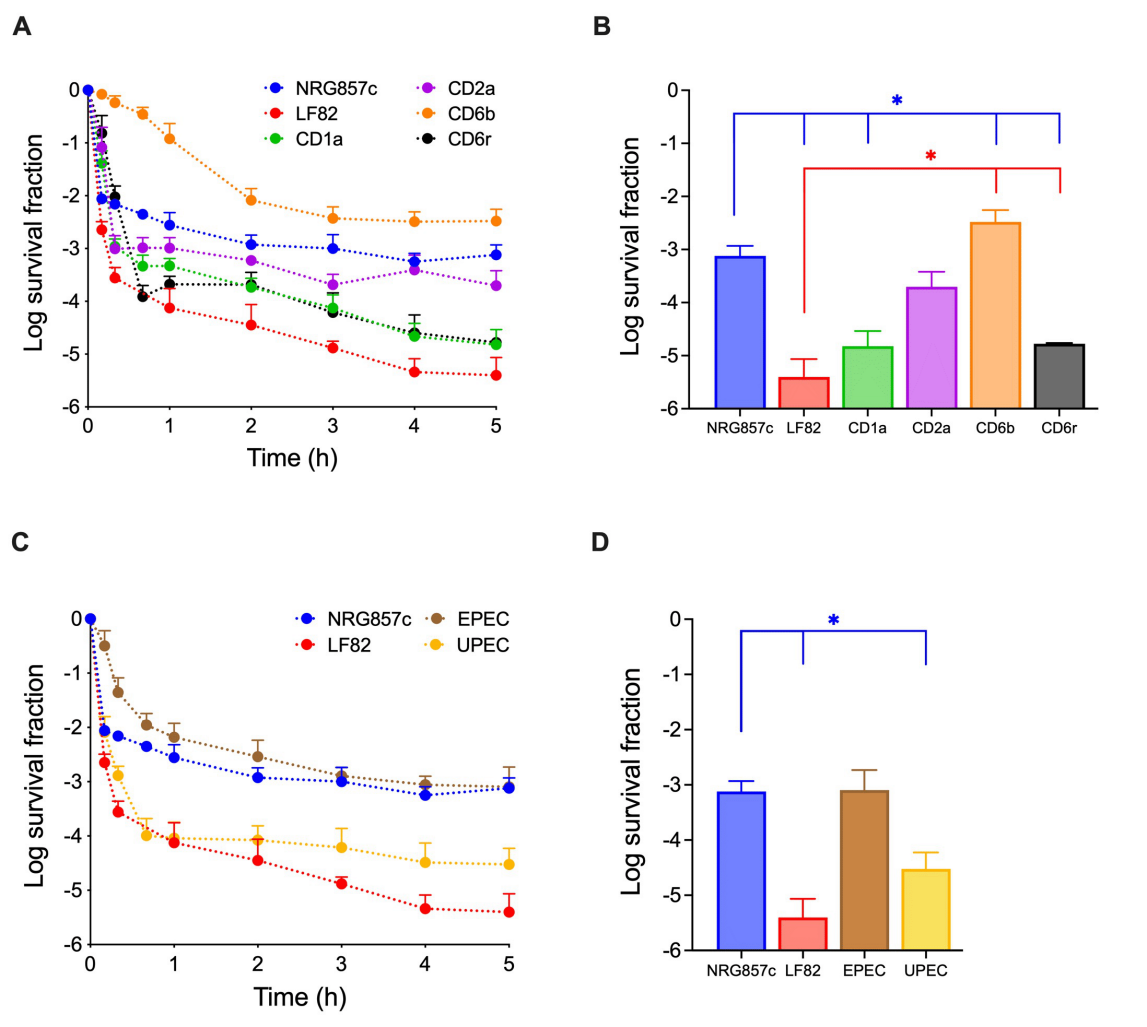
FIGURE 2: Time-killing curves of AIEC clinical isolates and *E. coli* pathotypes. **(A, B)** *E. coli* clinical isolates showing an AIEC phenotype, or **(C, D)** *E. coli* strains belonging to EPEC and UPEC pathotypes, were grown in LB broth, challenged with a 50-fold MIC of ciprofloxacin, and survival was monitored at the indicated times. The data for NRG857c and LF82 shown in Fig. 1A were included for reference purposes (blue and red lines, respectively). **(B, D)** Details of survival data at 5-hours post-treatment for each strain are graphed in A and C, respectively. Data points are mean values of at least three independent experiments, and standard deviations are represented by error bars above the mean. Student’s t-test was performed between NRG857c or LF82 and the other strains (*P < 0.05).

To determine whether the variability in persistence observed among AIEC reference strains also extends to other AIEC isolates, the survival of four clinical isolates exhibiting an AIEC phenotype, CD1a, CD2a, CD6b and CD6r 20, was tested following ciprofloxacin challenge (**Figure 2A** and **2B**). Our results revealed that 0.3% of CD6b bacterial population became persister cells after 5-hours of treatment, above the level observed for NRG857c (**Figure 2A** and **2B**). At the same time point, CD2a showed no significant difference compared to NRG857c, whereas CD1a and CD6r exhibited survival levels more similar to that of LF82 (**Figure 2A** and **2B**). While heterogeneity in persistence among clinical isolates is well recognized 21, our findings underscore that such heterogeneity also exists among isolates within the same pathotype. Moreover, we identified the presence of high-persistence strains within the AIEC pathotype as being concerning.

### The persistence levels of NRG857c resemble those of diarrheagenic *E. coli* than of extraintestinal strains

To determine whether the high-persistence observed in NRG857c is a unique feature of certain AIEC strains or also present in other *E. coli* pathotypes, killing curves of reference strains from the enteropathogenic *E. coli* (EPEC) and uropathogenic *E. coli* (UPEC) pathotypes were analyzed (**Figure 2C** and **2D**). Although AIEC strains are genetically more similar to extraintestinal pathogenic *E. coli* than to classical diarrheagenic strains 14, our results revealed that the persister levels of NRG857c are comparable to those of the EPEC reference strain, although significantly different from those of the UPEC reference strain (**Figure 2C** and **2D**). While 0.08% of the EPEC population became persister cells after 5-hour of treatment, only 0.003% of the UPEC population survived at the same time point, corresponding to a 30-fold lower persistence level than that observed for NRG857c.

Although other *E. coli* pathotypes should be investigated, our findings suggest that the high-persistence levels of NRG857c more closely resemble those of diarrheagenic *E. coli* than extraintestinal strains.

### NRG857c persistence levels remain unaltered after macrophage passage

**Figure 3:**
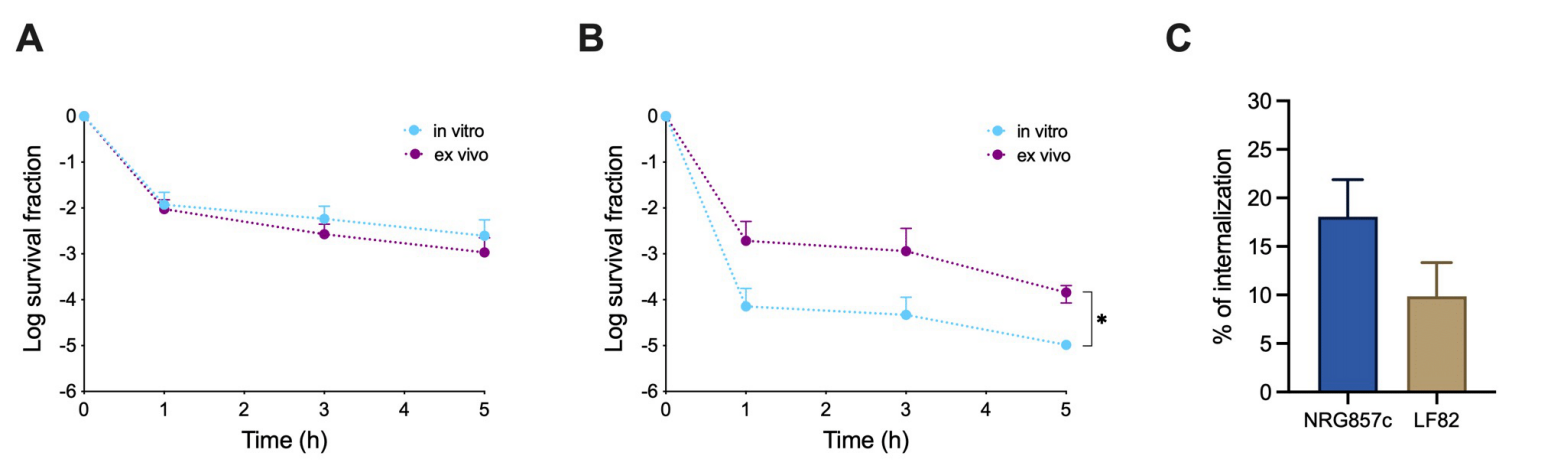
FIGURE 3: Killing curves of AIEC reference strains after macrophage passage. **(A)** Wild type NRG857c and **(B)** LF82 were cultivated up to OD_600nm_ 0.3 in LB broth (*in vitro*, light blue lines) or harvested after 30 min post-infection within macrophages (*ex vivo*, purple lines), challenged with ciprofloxacin, respectively, and survival was monitored at indicated time points. Ciprofloxacin was used at 50-fold MIC for NRG857c and 30-fold MIC for LF82. Data points are mean values of at least three independent experiments, and standard deviations are represented by error bars above the mean. Student’s t-test was performed between NRG857c or LF82 and the other strains (*P < 0.05). **(C)** Macrophage uptake observed 30 minutes post-infection.

A remarkable characteristic of AIEC is its capacity to survive and replicate within macrophages 13. *Salmonella* internalization into macrophages is necessary to trigger persister cell formation 3, a phenomenon similarly observed with *Mycobacterium tuberculosis* 22. Comparable behavior has been reported for LF82, where macrophage internalization induces a 50- to 500-fold increase in the formation of antibiotic-tolerant bacteria compared to those exponentially growing 8. Surprisingly, our findings revealed that macrophage internalization did not increase persister cell formation of NRG857c (**Figure 3A**). In contrast, our results showed increased persistence levels for LF82 following macrophage internalization (**Figure 3B**), consistent with previous findings 8. It is worth noting that with our experimental setup, NRG857c bacterial uptake by macrophages was 18% of the initial inoculum, which is comparable to the 20% reported by other authors 23, and with no significant differences to the internalization rate of LF82 (**Figure 3C**).

Altogether, our results suggest that NRG857c exhibits a high basal persistence level, which could be achieved under *in vitro* conditions and, unlike *Salmonella* or LF82, remains unaffected by the stress conditions found within macrophages.

### HtrA is not implicated in the high-persistence phenotype of NRG857c

**Figure 4:**
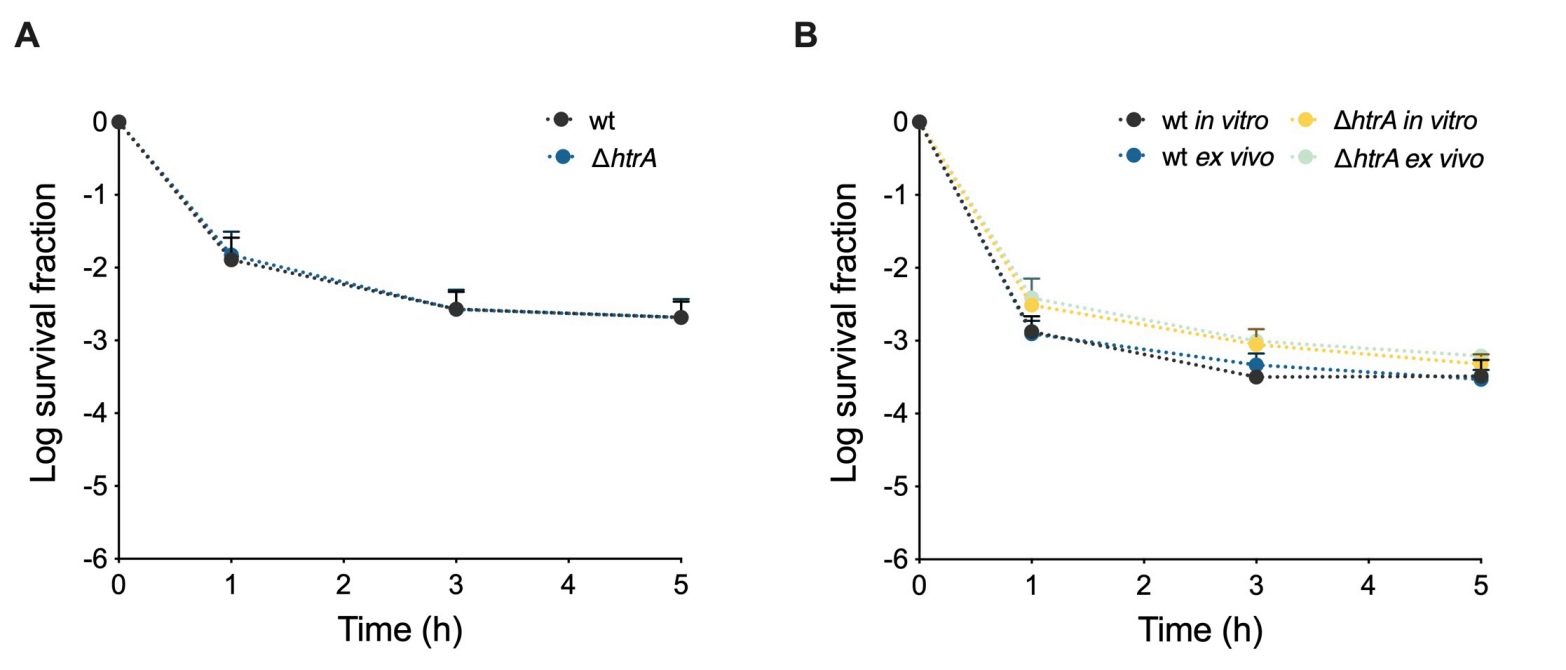
FIGURE 4: Killing curves of NRG87c and its *htrA* deletion mutants. **(A)** *In vitro* killing curves of wild type NRG857s and its *htrA* deletion mutant; the strains were grown in LB broth, challenged with 50-fold MIC of ciprofloxacin, and survival was monitored at indicated times points. **(B)** Killing curves of wild type NRG857c and its *htrA* deletion mutant after macrophage passage. Data points are mean values of three independent experiments, and standard deviation are represented by error bars above the mean. Student’s t-tests were performed between data from in vitro and ex vivo at 5-hours hours post-treatment, and no significant differences were observed.

HtrA is a protease that plays an essential role in the intra-macrophage lifestyle of AIEC 16. However, deletion of *htrA* did not impact the persistence levels of the AIEC reference strains (**Figure 4A** and S5). Notably, even after passage through macrophages, the NRG857c Δ*htrA* mutant showed no significant difference compared to the wild-type strain (**Figure 4B**). Overall, our findings suggest that HtrA is not involved in the persistence phenotype of AIEC, despite its crucial role in intramacrophage survival and stress response.

### Known high-persistence mutations are absent in NRG857c

Persistence is known to be a nonheritable phenotype. However, some high-persistence mutations have been described. The HipA7 variant contains two mutations, G22S and D291A, associated with a high-persistence phenotype 24. NRG857c HipA (WP_001125432) and its identical homologue in LF82 share 98.2% aminoacid identity with the K-12 HipA protein (NP_416024.1); however, both lack the key mutations characteristic of the HipA7 variant (Figure S6). Interestingly, some of their aminoacidic variations are also shared by HipA from UPEC CFT073 and EPEC E2348/69, which exhibit low and high persistence phenotypes, respectively (Figure S6, 2C and 2D). In addition to *hipA7*, the *hipA*(D88N) and *hipA*(P86L) variants -identified in clinical isolates or through laboratory screens- are also associated with a high-persistence phenotype 25. None of these variants are present in the HipA proteins of AIEC or other *E. coli* pathotypes analyzed in this study (Figure S6). Through our experiments, we exclude the possibility that NRG857c has acquired *hip*A mutations responsible for its high-persistence phenotype. This was confirmed by sequencing the *hipA* allele from surviving colonies after 3-hour of antibiotic treatment (Figure S7).

Although functional experiments are required, our results indicate that *hipA* does not appear to be necessary or sufficient for the high-persistence phenotype of NRG857c. Other chromosomal genetic factors are likely to be involved, as discussed below.

## DISCUSSION

For AIEC, the presence of persister cells, both in culture and intracellularly, had been demonstrated only for the LF82 strain 8. In this study, we expanded the antibiotic persister analysis to other AIEC strains and revealed that this pathotype comprises a heterogeneous population with different persister cell formation competencies. One of the most significant outcomes was the identification of high-persistence strains, which differ from the LF82 behavior.

Most research on AIEC has been carried out with LF82. However, despite it having genomic similarities with NRG857c 14, both strains have phenotypic differences. For instance, they induce a distinct inflammatory response in intestinal epithelial culture cells 26 and show different colonization capabilities in mouse models 27. Contrary to LF82, which did not colonize conventional mice for long periods, NRG857 can induce a persistent infection leading to chronic inflammation 27. As shown in this study, it is feasible to speculate that such phenotypic differences might be due to their dissimilarities in persister cell formation.

Main genetic differences between LF82 and NRG857c are localized in plasmids 14. However, though plasmid pO83_CORR carried by NRG857c has been linked to antimicrobial peptide resistance and colonization 28, our results discard its role in the persistence phenotype (**Figure 1B**). Whole-genome comparisons between NRG857c and LF82 14 revealed considerable sequence similarity and synteny. In addition to differences in plasmid content, 46 chromosomal genes were identified as unique to NRG857c and ten as unique to LF82. However, most NRG857c-specific genes were predicted to encode hypothetical proteins at the time. A reanalysis of these genes, based on the updated NRG857c reference genome (NCBI Reference Sequence NC_017634), led us to reveal that the number of chromosomal genes unique to this strain is limited to nine, the majority of which are located within genomic islands (Supplementary file S1). These NRG857c-specific genes represent plausible candidates underlying its persistence phenotype that need further investigation. Additionally, we cannot exclude the possibility that differences between strains may be attributed to SNPs variations or an epigenetic mechanism. For instance, SNPs can lead to the emergence of auxotrophic mutants with enhanced persistence 29, and recent findings indicate that epigenetic regulation plays a major role in the development of bacterial persistence 30,31.

Activation of stringent response through the (p)ppGpp synthase RelA and SpoT is important for *E. coli* persister cell formation 2, and there is evidence of its role in LF82 persistence 8. However, after several unsuccessful attempts to get *spoT*/*relA* deletion mutants in NRG857c or NRG857c(Cu), we were unable to reveal their role in the high-persistence phenotype. Furthermore, we discarded the role of *htrA* and *hipA* being responsible for the NRG857c high-persistence phenotype.

A loss-of-function mutation in the *ptsI* gene was recently associated with increased persister formation in relapsed *E. coli* isolates from bloodstream infections 32. Our *in silico* analysis revealed that NRG857c and LF82 encode identical *pstI* genes (Supplementary file S2), so gene functionality should be tested to elucidate their role in the high-persistence phenotype of NRG857c.

According to our findings, it is feasible to speculate a contribution of persister cells for NRG857c chronic colonization. Still, since genetic and phenotypic heterogenicity exists among AIEC isolates, coupled with the reduced AIEC strains analyzed here, it needs further clarification whether the high-persistence phenotype is a common characteristic among the pathotypes or a particularity of specific strains. Surprisingly, NRG857c persister cells seem to consist of a high basal population fraction which raises the intriguing question, is there a maximum persistence level that bacteria could reach?

Our study expands the known diversity among AIEC strains and underlines the importance of further studies examining the role of AIEC persisters on ongoing CD, symptom relapse, and response to antibiotic treatment. Particularly, our observation of high-persistence AIEC strains raises concerns over the effectiveness of current antibiotic therapy to treat CD patients, as antibiotics could potentiate AIEC infection and expansion 33.

## MATERIAL AND METHODS

### Bacterial strains and growth conditions

Bacterial strains used in this study are described at **Table 2**. Bacteria were grown routinely in Luria-Bertani Lennox (LB) broth (BD Difco) at 37°C with shaking at 170 rpm. When needed, 1,5% p/v agar (LB-agar) or 15 µg/mL gentamicin (Sigma) was added.

**Table 2:** Bacterial strains and plasmids used in this work.

**Strain/plasmid**	**Comments**	**Source**
LF82	AIEC reference strain	Gift from Olivier Espéli
LF82 Δ*htrA*::Km	LF82 *htrA* deletion mutant	Gift from Olivier Espéli
NRG857c	AIEC reference strain	Gift from Alfredo Torres
NRG857c Δ*htrA*::Gm	NRG857c* htrA* deletion mutant	This work
NRG857c(Cu)	NRG857c strain cured of its resistance plasmid [19]	Gift from Alfredo Torres
H10407	Enterotoxigenic *E. coli* (ETEC) reference strain	Lab collection
E2348/69	Enteropathogenic *E. coli* (EPEC) reference strain	Lab collection
EI-34	Enteroinvasive *E. coli* (EIEC) reference strain	Lab collection
F-1845	Diffusely adherent *E. coli* (DAEC) reference strain	Lab collection
CFT073	Uropathogenic *E. coli* (UPEC) reference strain	Lab collection
CD1a	Clinical isolated with AIEC phenotype 20	Lab collection
CD2a	Clinical isolated with AIEC phenotype 20	Lab collection
CD6b	Clinical isolated with AIEC phenotype 20	Lab collection
CD6r	Clinical isolated with AIEC phenotype 20	Lab collection
pKD46_Km	Recombinase-expressing plasmid 35	Gift from Brian Coombes
pGP-Tn7-Gm	Gentamicin resistance cassette template plasmid 36	Gift from Charles Dozois

### Polymerase chain reaction (PCR)

PCR reactions were done using the SapphireAmp Fast PCR Master Mix (Takara), 0.2 µM of oligonucleotides (**Table 3**) and 20 ng of pGP-Tn7-Gm or colony lysates as template. The cycling program consisted of an initial denaturation at 94°C per 1 min, 30 cycles of 98°C per 5 sec, 55°C per 5 sec, and 72°C per 20 sec, followed by a final extension at 72°C per 2 min.

**Table 3:** Oligonucleotides used in this study.

**Oligonucleotide**	**Sequence (5’to 3’)**
koHtrA-40_G1.2	GCAATTTTGCGTTATCTGTTAATCGAGACTGAAATACATGGGACGATCGAATTGGGGATC
koHtrA-40_G2.2	AGGAAGGGGTTGAGGGAGATTACTGCATTAACAGGTAGATATCCACTAGTGAGCTCATGC
Up_HtrA-F	GGCCGTAGAACAATAACCAG
Down_HtrA-R	TCGTGCAATTCACCAATACG

### *htrA* deletion mutant construction

The AIEC strain NRG857c Δ*htrA* mutant was generated via Lambda-Red recombination 34 using the pKD46_Km recombinase-expressing plasmid 35. Oligonucleotides koHtrA-40_G1.2 and koHtrA-40_G2.2 were used to amplify the gentamicin resistance cassette using pGP-Tn7-Gm 36 as a template. Transformants carrying pKD46_Km were transformed with the PCR product and spread onto LB-agar supplemented with gentamicin. Colonies were screened by colony PCR using oligonucleotides Up_HtrA-F and Down_HtrA-R, and gene disruption was confirmed by Sanger sequencing (Macrogen-Chile).

### Minimum Inhibitory Concentrations (MIC)

Susceptibilities to ciprofloxacin (Sigma) were determined by the broth microdilution method in Mueller-Hinton broth (BD Difco) with inocula of 5x10^5^ CFU/mL, according to CLSI M07-A10 guidelines 37. Microplates were incubated statically overnight at 37°C, and MIC values were determined as the lowest antibiotic concentration that inhibited growth. All MIC values were calculated from three independent experiments, involving three replicates each.

### Time-killing curves

Overnight cultures were inoculated from frozen glycerol stocks into LB broth and grown overnight. Fresh LB broth was inoculated at a starting OD_600nm_ of 0.03 and grown until it reached the early exponential growth phase (OD_600nm_ 0.3-0.4). Ciprofloxacin was added to 20-100-fold MIC to each culture and grown up to 5-hours. Samples were taken at several time points after the antibiotic challenge, serially diluted in phosphate-buffered saline (PBS, Merck), and plated on LB-agar without antibiotics. After incubation, CFU/mL were determined, and the survival ratio (regarding the number of CFU/mL at a given time to the number of CFU/mL at the treatment time) was graphed as a function of time. Time-killing curves were performed in biological triplicate.

### Macrophage-induced persisters

The J774.A1 murine macrophage cell line (ATCC TIB-67) was maintained in high-glucose Dulbecco’s Modified Eagle (DMEM) medium (HyClone^TM^ Cytiva) supplemented with 10% fetal bovine serum (HyClone^TM^ Cytiva) and penicillin/streptomycin (Corning). Cells were grown at 37°C with 5% CO_2_ with regular media changes. For infection assays, macrophages were seeded at 9.5x10^5^ cells per well in a 6-well plate (SPL Life Sciences) 20-24 hours prior to infection. Bacteria were grown in LB broth until the early exponential phase and then diluted in non-supplemented DMEM medium to infect macrophages at a multiplicity of infection of 10. After 10 min of centrifugation at 900xg and a 20 min incubation period at 37°C with 5% CO_2_ (30 min infection in total), infected macrophages were washed with PBS and lysed with 0.1% Triton X-100 (Merck). Intracellular bacteria were collected by centrifugation at 14,000xg per 2 min and resuspended in fresh LB broth. The antibiotic was added to the culture, and the time-killing curve protocol was followed as above. The uptake values (internalization) were determined 30 minutes post-infection and are expressed as a percentage of the initial inoculum used for infection. The survival of the macrophage-exposed population (ex vivo persisters) was compared to the survival of bacteria used as inoculum for macrophage infection (in vitro persisters).

### Bioinformatic analysis

Nucleotides and protein sequences were obtained from the NCBI database. *E. coli *MG1655 HipA protein sequence (NP_416024.1) was used as a query to search by tblastn at NCBI. Multiple sequence alignments were done using the Clustal Omega (1.2.4) program available at the EMBL-EBI website 38.

### Statistical analysis

Statistical differences were determined using a two-tailed Student *t*-Test on the means of at least three independent experiments, using GraphPad Prism 10 Version 10.3.0. Differences were considered statistically significant when P < 0.05.

## CONFLICT OF INTEREST

The authors have declared that no conflict of interest exists with this study.

## SUPPLEMENTAL MATERIAL

Click here for supplemental data file.

All supplemental data for this article are available online at www.microbialcell.com/researcharticles/2025a-perez-villalobos-microbial-cell/.
