# Supplementary material for: Persistence phenotype of adherent-invasive Escherichia coli in response to ciprofloxacin, revealing high-persistence strains
Source: Microb Cell. 2025 Jul 11;12:173–81. doi: 10.15698/mic2025.07.854 (PMC12321266; doi:10.15698/mic2025.07.854)
Supplement: Supplementary file 1 [file mic-12-173-s01.pdf]

Supplemental Material

Persistence phenotype of adherent-invasive *Escherichia coli* in response to ciprofloxacin, revealing high-persistence strains

Valeria Pérez-Villalobos[1], Roberto Vidal[2], Marcela A. Hermoso[3,4] and Paula Bustamante[1,*]

[1] Molecular and Cellular Microbiology Laboratory, Instituto de Ciencias Biomédicas, Facultad de Ciencias de la Salud, Universidad Autónoma de Chile, Chile. [2] Programa de Microbiología y Micología, Instituto de Ciencias Biomédicas, Facultad de Medicina, Universidad de Chile, Chile. [3] Laboratory of Innate Immunity, Program of Immunology, Institute of Biomedical Sciences, Faculty of Medicine, Universidad de Chile, Santiago, Chile. [4] Department of Gastroenterology and Hepatology, University Medical Center Groningen, Groningen, Netherlands.

⋆ Corresponding Author:
Paula Bustamante. Molecular and Cellular Microbiology Laboratory, Instituto de Ciencias Biomédicas, Facultad de Ciencias de la Salud, Universidad Autónoma de Chile. Av. del Valle 534, Level 3, Huechuraba, Santiago, 8581151, Chile;
E-mail: paula.bustamante@uautonoma.cl

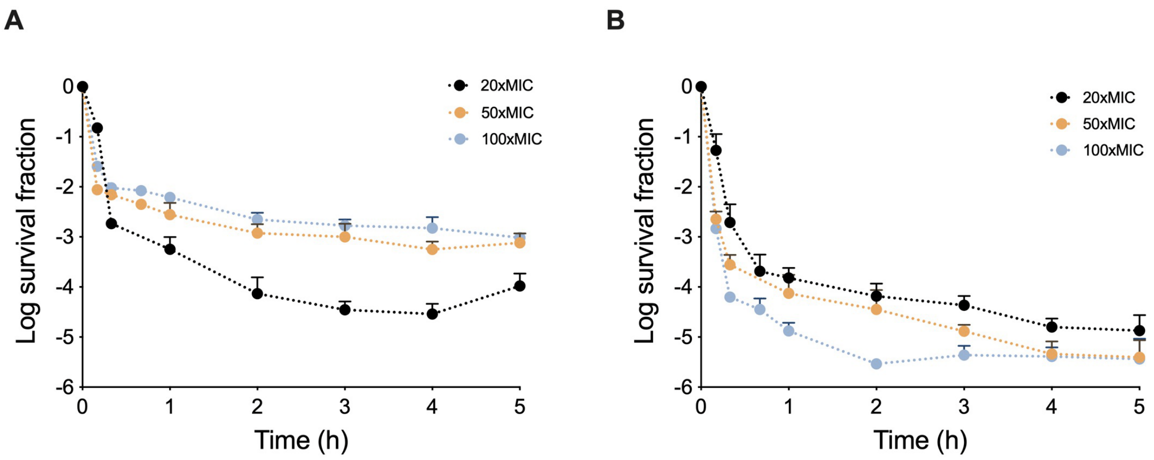

Supplemental FIGURE S1 ● Time-killing curves of reference AIEC strains exposed to varying concentrations of antibiotic. (A) NRG857c and (B) LF82 strains were grown in LB broth, challenged with 20-, 50- and 100-fold MIC of ciprofloxacin, and survival was monitored at indicated times. Data points are mean values of three independent experiments, and standard deviation are represented by error bars above the mean.

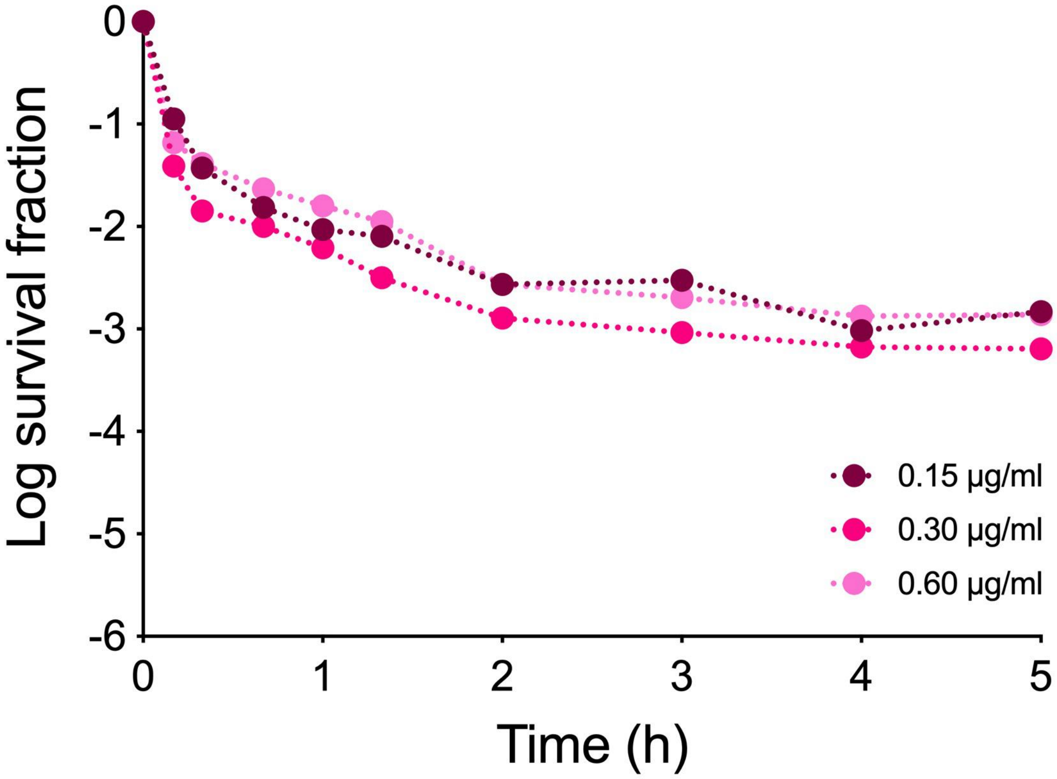

Supplemental FIGURE S2 ● **Time-killing curve of NRG857c in MOPS-minimal medium.** The NRG857c strain was grown in a MOPS-based medium supplemented with 0.4% glucose until OD_{600nm} 0.3, then challenged with ciprofloxacin at 0.15, 0.30, and 0.60 µg/mL, and survival was monitored at indicated times.

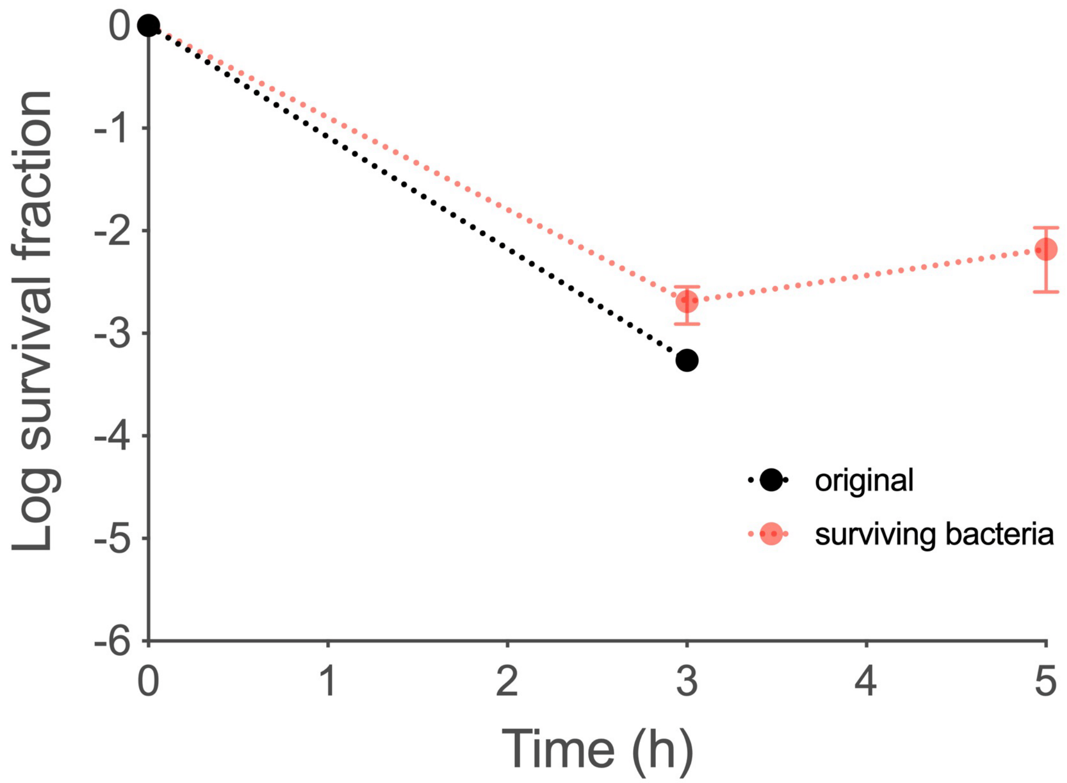

Supplemental FIGURE S3 ● **Time-killing curve of NRG857c surviving bacteria.** Survival bacteria recovered after 3-hours post-ciprofloxacin challenge (50-fold MIC; black line), were grown and treated again with antibiotic at the same original conditions, and survival fraction was calculated at different time points (orange line). Data points are the mean values of three independent experiments.

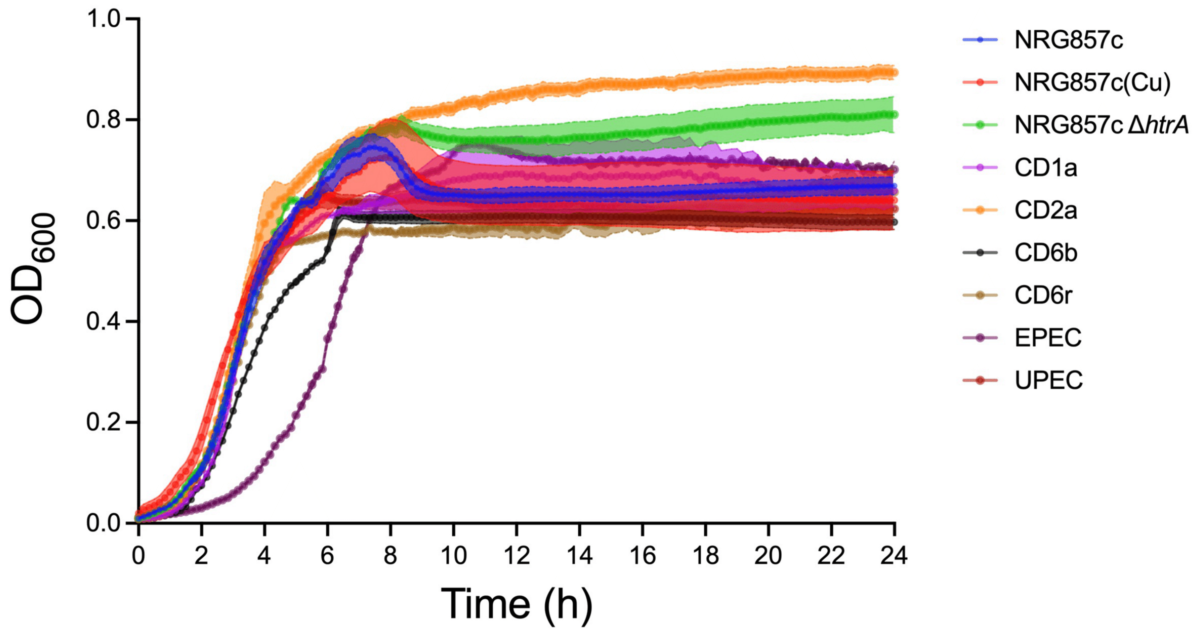

Supplemental FIGURE S4 ● MOPS-minimal medium growth curves of *E. coli* strains used in this study. LF82 and LF82 Δ*htrA* were not included, as they were unable to grow in this minimal medium.

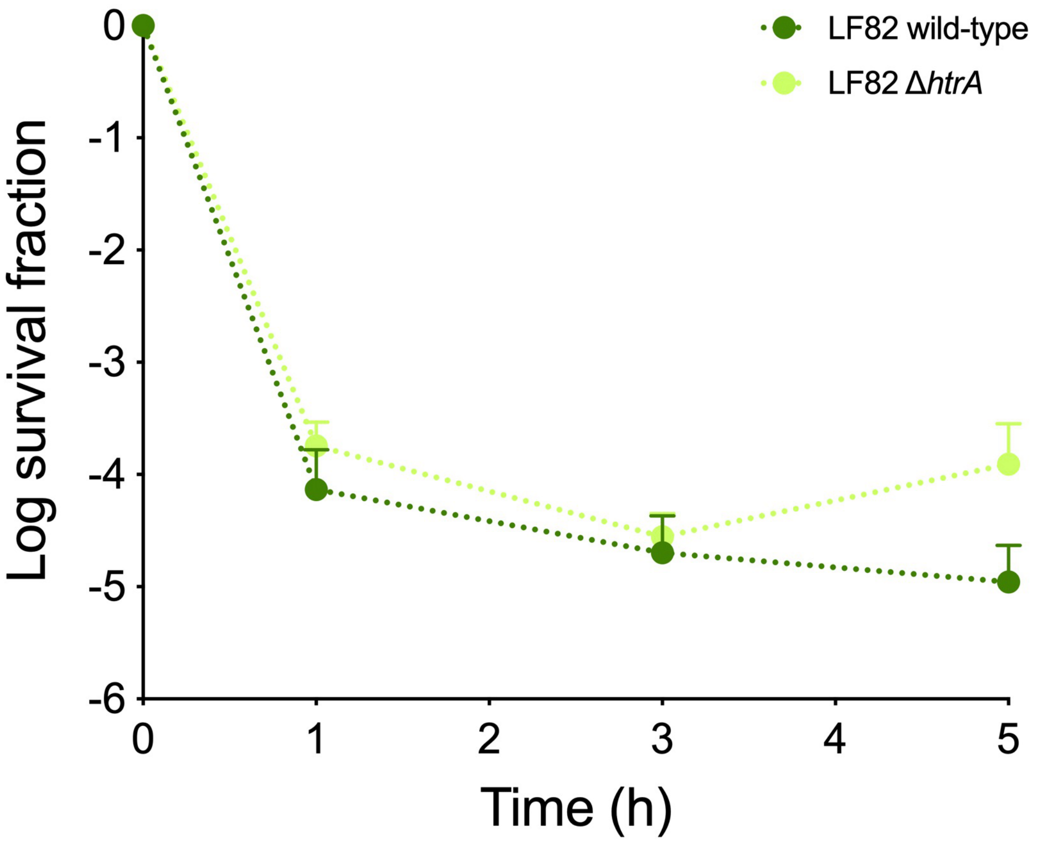

Supplemental FIGURE S5 ● **Killing curves of LF82 and its *htrA* deletion mutant *in vitro*.** The strains were grown in LB broth, challenged with 30-fold MIC of ciprofloxacin, and survival was monitored at indicated times points. Data points are mean values of three independent experiments, and standard deviation are represented by error bars above the mean. Student's t-test was performed on the data 5-hours post treatment, and no significant differences were observed.

```
HipA_MG1655     MPKLVTWMNNQRVGELTKLANGAHTFKYAPEWLASRYARPLSLSLPLQRGNITSDAVFNF     60
NRG857c         MPKLVTWMNNQRVGELTKLANGAHTFKYAPEWLASRYARPLSLSLPLQRGNITSDAVFNF     60
LF82            MPKLVTWMNNQRVGELTKLANGAHTFKYAPEWLASRYARPLSLSLPLQRGNITSDAVFNF     60
EPEC            MPKLVTWMNNQRVGELTKLANGAHTFKYAPEWLASRYARPLSLSLPLQRGNITSDAVFNF     60
UPEC            MPKLVTWMNNQRVGELTKLANGAHTFKYAPEWLASRYARPLSLSLPLQRGNITSDAVFNF     60
ETEC            MPKLVTWMNNQRVGELTKLANGAHTFKYAPEWLASRYARPLSLSLPLQRGNITSDAVFNF     60
                ************************************************************

HipA_MG1655     FDNLLPDSPIVRDRIVKRYHAKSRQPFDLLSEIGRDSVGAVTLIPEDETVTHPIMAWEKL     120
NRG857c         FDNLLPDSPIVRDRIVKRYHAKSRQPFDLLSEIGRDSVGAVTLIPEDETVTCPIMAWEKL     120
LF82            FDNLLPDSPIVRDRIVKRYHAKSRQPFDLLSEIGRDSVGAVTLIPEDETVTCPIMAWEKL     120
EPEC            FDNLLPDSPIVRDRIVKRYHAKSRQPFDLLSEIGRDSVGAVTLIPEDETVTCPIMAWEKL     120
UPEC            FDNLLPDSPIVRDRIVKRYHAKSRQPFDLLSEIGRDSVGAVTLIPEDETVTCPIMAWEKL     120
ETEC            FDNLLPDSPIVRDRIVKRYHAKSRQPFDLLSEIGRDSVGAVTLIPEDETVTHPIMAWEKL     120
                *************************************************** ********

HipA_MG1655     TEARLEEVLTAYKADIPLGMIREENDFRISVAGAQEKTALLRIGNDWCIPKGITPTTHII     180
NRG857c         TEARLEEVLTAYKADIPLGMIREENDFRISVAGAQEKTALLRIGNDWCIPKGITPTTHII     180
LF82            TEARLEEVLTAYKADIPLGMIREENDFRISVAGAQEKTALLRIGNDWCIPKGITPTTHII     180
EPEC            TEARLEEVLTAYKADIPLGMIREENDFRISVAGAQEKTALLRIGNDWCIPKGITPTTHII     180
UPEC            TEARLEEVLTAYKADIPLGMIREENDFRISVAGAQEKTALLRIGNDWCIPKGITPTTHII     180
ETEC            TEARLEEVLTAYKADIPLGMIREENDFRISVAGAQEKTALLRIGNDWCIPKGITPTTHII     180
                ************************************************************

HipA_MG1655     KLPIGEIRQPNATLDLSQSVDNEYYCLLLAKELGLNVPDAEIIKAGNVRALAVERFDRRW     240
NRG857c         KLPIGEIRQPNATLDLSQSVDNEYYCLLLAKELGLNVPDAEIIKAGRVRALAVKRFDRRW     240
LF82            KLPIGEIRQPNATLDLSQSVDNEYYCLLLAKELGLNVPDAEIIKAGRVRALAVKRFDRRW     240
EPEC            KLPIGEIRQPNATLDLSQSVDNEYYCLLLAKELGLNVPDAEIIKAGRVRALAVERFDRRW     240
UPEC            KLPIGEIRQPNATLDLSQSVDNEYYCLLLAKELGLNVPDAEIIKAGRVRALAVKRFDRRW     240
ETEC            KLPIGEIRQPNAMLDLSQSVDNEYYCLLLAKELGLNVPDAEIIKAGNVRALAVERFDRRW     240
                ************ *********************************.******:******

HipA_MG1655     NAERTVLLRLPQEDMCQTFGLPSSVKYESDGGPGIARIMAFLMGSSEALKDRYDFMKFQV     300
NRG857c         NTERTVLLRLPQEDMCQTFGLPSSVKYESDGGPGIAQIMAFLMGSSEALKDRYDFMKFQV     300
LF82            NTERTVLLRLPQEDMCQTFGLPSSVKYESDGGPGIAQIMAFLMGSSEALKDRYDFMKFQV     300
EPEC            NTERTVLLRLPQEDMCQTFGLPSSVKYESDGGPGIAQIMAFLMGSSEALKDRYDFMKFQV     300
UPEC            NTERTVLLRLPQEDMCQTFGLPSSVKYESDGGPGIAQIMAFLMGSSEALKDRYDFMKFQV     300
ETEC            NAERTVLLRLPQEDMCQTFGLPSSVKYESDGGPGIARIMAFLMGSSEALKDRYDFMKFQV     300
                *:**********************************:***********************

HipA_MG1655     FQWLIGATDGHAKNFSVFIQAGGSYRLTPFYDIISAFPVLGGTGIHISDLKLAMGLNASK     360
NRG857c         FQWLIGATDGHAKNFSVFIQAGGSYRLTPFYDIISAFPVLGGTGIHISDLKLAMGLNASK     360
LF82            FQWLIGATDGHAKNFSVFIQAGGSYRLTPFYDIISAFPVLGGTGIHISDLKLAMGLNASK     360
EPEC            FQWLIGATDGHAKNFSVFIQAGGSYRLTPFYDIISAFPVLGGTGIHISDLKLAMGLNASK     360
UPEC            FQWLIGATDGHAKNFSVFIQAGGSYRLTPFYDIISAFPVLGGTGIHISDLKLAMGLNASK     360
ETEC            FQWLIGATDGHAKNFSVFIQAGGSYRLTPFYDIISAFPVLGGTGIHISDLKLAMGLNASK     360
                ************************************************************

HipA_MG1655     GKKTAIDKIYPRHFLATAKVLRFPEVQMHEILSDFARMIPAALDNVKTSLPTDFPENVVT     420
NRG857c         GKKTAIDKIYPRHFLATAKVLKFPEVQMHEILSDFARMIPAALDNVKTSLPTDFPENVVT     420
LF82            GKKTAIDKIYPRHFLATAKVLKFPEVQMHEILSDFARMIPAALDNVKTSLPTDFPENVVT     420
EPEC            GKKTAIDKIYPRHFLATAKVLKFPEVQMHEILSDFARMIPAALDNVKTSLPTDFPENVVT     420
UPEC            GKKTAIDKIYPRHFLATAKVLKFPEVQMHEILSDFARMIPAALDNVKNSLPTDFPENVVT     420
ETEC            GKKTAIDKIYPRHFLATAKVLRFPEVQMHEILSDFARMIPAALDNVKTSLPTDFPENVVT     420
                *********************:*************************.************

HipA_MG1655     AVESNVLRLHGRLSREYGSK       440
NRG857c         AVETNVLRLHGRLSREYGIK       440
LF82            AVETNVLRLHGRLSREYGIK       440
EPEC            AVETNVLRLHGRLSREYGIK       440
UPEC            AVETNVLRLHGRLSREYGIK       440
ETEC            AVESNVLRLHGRLS------       434
                ***:**********
```

Supplemental FIGURE S6 ● **Aminoacid alignment of HipA sequences from reference *E. coli* pathotypes.** Residues that differ from the canonical HipA from *E. coli* MG1655 are highlighted in black. G22 and D291 residues, which show variants in HipA7, are highlighted in red. D88 and P86 residues, which exhibit variants associated with high-persistence phenotypes in patient samples or laboratory screens, are highlighted in yellow. HipA sequences accession numbers: NP_416024.1 (MG1655), WP_001125432 (NRG857c), WP_001125432 (LF82), CAS09186 (EPEC E2348/69), WP_001125431 (UPEC CFT073) and WP_001125437 (ETEC H10407).

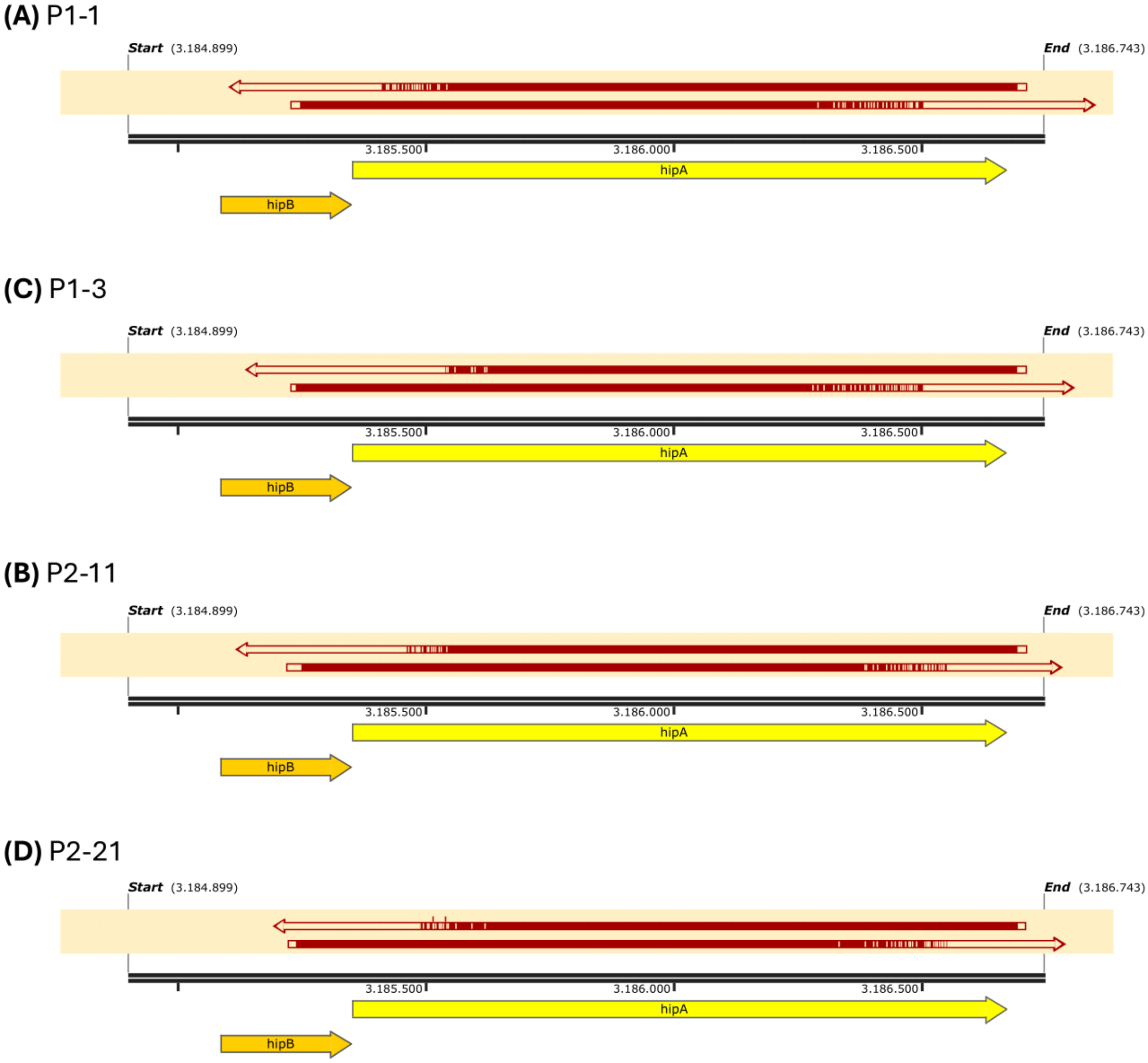

Supplemental FIGURE S7 ● **Alignment of *hipA* gene sequences from persister cells to the reference sequence of the NRG857c parental strain.** *hipA* genes were PCR amplified from bacterial colonies (P1-1, P1-3, P2-11 and P2-21) recovered after 3 hour of antibiotic treatment; PCR products were sequenced (Macrogen, Chile) using forward (5’-AGCAGGCGACAATTTCCAAT-3’) and reverse (5’-AAGGGAGAATCCAGTCGTTG-3’) primers to cover the entire *hipA* gene, and the resulting sequences were align to the NRG857c *hipA* gene as reference (NRG857_RS07640, yellow arrow) using the SnapGene software version 8.0.2. Burgundy coloring in the arrows indicates a perfect match with the reference sequence.

Supplemental TABLE S1 ● **Ciprofloxacin MIC values of NRG857c survival bacteria and their parental strain.** MIC represents mean values and SD of three independent experiments.

Strain	MIC (μg/mL) ± SD
Parental NRG857c	0.0078 ± 0.0000
Survival bacteria	0.0078 ± 0.0000

Supplementary File S1

Supplementary file S1. Chromosomal genes unique to NRG857c identified by Nash et al., 2010 and their homologues in LF82.

			NCBI Reference Sequence: NC_017634.1			blastn against LF82 (NC_011993.1)		
Old locus tag #	Coordenates	Product	New locus tag	CDS	Comments	blastn identity	location	locus tag
NRG857_00010	complement(108..344)	hypothetical protein	NRG857_RS00005	WP_032148145.1	reannotates as *thrL* gene; *thr* (threonine biosynthesis) operon leader peptide	100%	4,772,957..4,773,028	thrL
NRG857_01160	263,663..263,773	hypothetical protein			not annotated in the NCBI Reference Sequence	100%	263,321..263,431	no gene annotated
NRG857_03620	794,687..794,809	hypothetical protein			not annotated in the NCBI Reference Sequence	100%	798,887..799,009	no gene annotated
NRG857_04685	complement(1,012,384..1,012,542)	hypothetical protein			not annotated in the NCBI Reference Sequence	100%	1,015,597..1,015,755	no gene annotated
NRG857_04720	1,015,059..1,015,553	DNA packaging protein gp3 (small subunit)	NRG857_RS04825	WP_000729920.1	DNA-packaging protein; within genomic island GI-7	No significant similarity found		
NRG857_04725	1,015,531..1,017,030	DNA packaging protein gp2 (large subunit)	NRG857_RS04830	WP_000417851.1	within genomic island GI-7	No significant similarity found		
NRG857_04745	1,021,461..1,021,739	hypothetical protein	NRG857_RS04850	WP_014640124.1	hypothetical protein	No significant similarity found		
NRG857_04790	complement(1,029,734..1,029,943)	hypothetical protein	NRG857_RS04900	WP_001036008.1	within genomic island GI-8	No significant similarity found		
NRG857_04800	1,030,297..1,030,437	hypothetical protein	NRG857_RS04910	WP_000865491.1	Arc family DNA-binding protein; within genomic island GI-8	No significant similarity found		
NRG857_04975	1,071,977..1,072,081	hypothetical protein			not annotated in the NCBI Reference Sequence	100%	1,074,115..1,074,219	no gene annotated
NRG857_05587	1,181,850..1,181,999	hypothetical protein	NRG857_RS05710	WP_014640128.1		100%	1,184,167..1,184,316	LF82_RS25585
NRG857_05720	complement(1,201,037..1,201,573)	hypothetical protein	NRG857_RS05850	WP_001049527.1	hypothetical protein	No significant similarity found		
NRG857_05775	1,208,278..1,208,472	hypothetical protein	NRG857_RS05900	WP_000453554.1	hypothetical protein	No significant similarity found		
NRG857_05780	complement(1,208,536..1,209,084)	hypothetical protein	NRG857_RS05905	WP_000389900.1	HEPN domain-containing protein	No significant similarity found		
NRG857_05910	complement(1,232,031..1,232,156)	hypothetical protein	NRG857_RS06035	WP_001445545.1	hypothetical protein	100%	1,223,273..1,223,398	LF82_RS25590
NRG857_06905	1,432,042..1,432,161	hypothetical protein	NRG857_RS07075, pseudo		metal ABC transporter permease	100%	1,423,285..1,423,404	LF82_RS25635, pseudo
NRG857_07205	1,501,282..1,501,761	hypothetical protein	NRG857_RS07375	WP_000594486.1	DUF4279 domain-containing protein	100%	1,492,468..1,492,947	LF82_RS07480
NRG857_07380	complement(1,538,912..1,539,055)	hypothetical protein			not annotated in the NCBI Reference Sequence	100%	1,530,100..1,530,243	no gene annotated
NRG857_07460	complement(1,556,576..1,556,827)	hypothetical protein	NRG857_RS07610, pseudo		NRG857_07460 sequence overlaps with a psudogene in the NCBI Reference S	100%	1,547,764..1,548,015	overlap with LF82_RS07735, pseudogen
NRG857_07845	complement(1,626,945..1,628,432)	hypothetical protein	NRG857_RS08035	WP_000354584.1	P-loop ATPase, Sll1717 family; within genomic island GI-16	No significant similarity found		
NRG857_08000	1,655,897..1,656,232	hypothetical protein	*asr*	WP_001362115.1	reannotates as asr gene;	100%	1,648,833..1,649,168	asr
NRG857_09525	complement(1,956,970..1,957,146)	hypothetical protein	*yecR*	WP_001237881.1	sequence overlaps with yecR gene; YecR-like lipofamily protein	100%	1,949,905..1,950,081	overlaps with *yecR*
NRG857_10005	2,063,167..2,063,589	hypothetical protein	NRG857_RS10220	WP_001164104.1	hypothetical protein	100%	2,056,104..2,056,526	LF82_RS10405
NRG857_13983	2,956,289..2,956,483	hypothetical protein	*ygdT*	WP_000953278.1	sequence includes ygdT gene	100%	2,982,066..2,982,260	includes *ygdT*
NRG857_16210	complement(3,435,648..3,435,773)	hypothetical protein			not annotated in the NCBI Reference Sequence	100%	3,461,418..3,461,543	no gene annotated
NRG857_16525	complement(3,482,618..3,482,857	hypothetical protein	*gspM* , pseudo		sequence is inside the gspM pseudo gene	100%	3,508,389..3,508,628	inside *gspM* pseudogene
NRG857_17923	3,797,845..3,798,039	hypothetical protein			not annotated in the NCBI Reference Sequence	100%	3,822,785..3,822,979	no gene annotated
NRG857_18295	complement(3,875,700..3,875,900)	hypothetical protein			not annotated in the NCBI Reference Sequence	100%	3,900,641..3,900,841	no gene annotated
NRG857_19025	4,038,499..4,038,609	hypothetical protein	*tssD* , pseudo	type VI secretion sys	sequence is inside the tssD pseudo gene	100%	4,063,440..4,063,550	inside the *tssD* pseudogene
NRG857_19303	complement(4,100,840..4,101,073)	conserved hypothetical protein			not annotated in the NCBI Reference Sequence	100%	4,125,782..4,126,015	no gene annotated
NRG857_20660	4,410,529..4,410,690	hypothetical protein				100%	4,435,471..4,435,632	no gene annotated
NRG857_20765	4,434,320..4,434,412	hypothetical protein			not annotated in the NCBI Reference Sequence	100%	4,459,262..4,459,354	no gene annotated
NRG857_20780	4,436,266..4,436,523	hypothetical protein	NRG857_RS21255	WP_001280704.1	hypothetical protein	100%	4,461,208..4,461,465	LF82_RS21975
NRG857_20845	4,447,445..4,447,555	hypothetical protein			not annotated in the NCBI Reference Sequence	100%	4,472,387..4,472,497	no gene annotated
NRG857_20950	complement(4,466,702..4,466,914)	hypothetical protein	NRG857_RS21420	WP_001058146.1	hypothetical protein	100%	4,491,650..4,491,862	LF82_RS22140
NRG857_20955	complement(4,466,938..4,467,267)	hypothetical protein	NRG857_RS21425	WP_001291841.1	hypothetical protein	100%	4,491,886..4,492,215	LF82_RS22145
NRG857_20965	4,468,624..4,468,761	hypothetical protein			not annotated in the NCBI Reference Sequence	100%	4,493,572..4,493,709	no gene annotated
NRG857_20975	4,469,949..4,470,230	hypothetical protein	NRG857_RS21440	WP_000184919.1	hypothetical protein	100%	4,494,897..4,495,178	LF82_RS22160
NRG857_20980	4,470,234..4,470,428	hypothetical protein	NRG857_RS21445	WP_000701593.1	DUF7706 family protein	100%	4,495,182..4,495,376	LF82_RS22165
NRG857_20985	4,470,504..4,470,707	hypothetical protein	NRG857_RS21450	WP_000202245.1	hypothetical protein	100%	4,495,452..4,495,655	LF82_RS22170
NRG857_21010	4,474,203..4,474,427	hypothetical protein	NRG857_RS21475	WP_014640264.1	hypothetical protein	100%	4,499,151..4,499,375	LF82_RS22195
NRG857_21345	4,539,339..4,539,458	hypothetical protein			not annotated in the NCBI Reference Sequence	99.17%	4,564,287..4,564,406	no gene annotated
NRG857_21715	4,623,371..4,623,655	hypothetical protein	NRG857_RS22200	WP_001338063.1	sequence is inside the NRG857_RS22200 gene; hypothetical protein	100%	4,648,319..4,648,603	inside gene LF82_RS22930
NRG857_21850	4,656,469..4,656,693	hypothetical protein	NRG857_RS22335	WP_001204018.1	hypothetical protein	100%	4,681,417..4,681,641	LF82_RS23090
NRG857_22125	4,718,153..4,718,314	hypothetical protein	*ytjA*	WP_000490275.1	DUF1328 domain-containing protein	100%	4,743,101..4,743,262	*ytjA*
NRG857_22150	4,722,939..4,723,040	hypothetical protein			not annotated in the NCBI Reference Sequence	100%	4,747,887..4,747,988	no gene annotated

#, Whole-genome comparison by Nash et al., 2010 was done using the GenBank: CP001855.1 NRG857c genome sequence, which later was updated to NC_011993.1.

Supplementary File S2

```
ptsI_MG1655      ATGATTTCAGGCATTTTAGCATCCCCGGGTATCGCTTTCGGTAAAGCTCTGCTTCTGAAA   60
ptsI_NRG857c     ATGATTTCAGGCATTTTAGCATCCCCGGGTATCGCTTTCGGTAAAGCTCTGCTTCTGAAA   60
ptsI_LF82        ATGATTTCAGGCATTTTAGCATCCCCGGGTATCGCTTTCGGTAAAGCTCTGCTTCTGAAA   60
                 ************************************************************

ptsI_MG1655      GAAGACGAAATTGTCATTGACCGGAAAAAAATTTCTGCCGACCAGGTTGATCAGGAAGTT   120
ptsI_NRG857c     GAAGACGAAATTGTCATTGACCGGAAAAAAATTTCTGCCGACCAGGTTGATCAGGAAGTT   120
ptsI_LF82        GAAGACGAAATTGTCATTGACCGGAAAAAAATTTCTGCCGACCAGGTTGATCAGGAAGTT   120
                 ************************************************************

ptsI_MG1655      GAACGTTTTCTGAGCGGTCGTGCCAAGGCATCAGCCCAGCTGGAAACGATCAAAACGAAA   180
ptsI_NRG857c     GAACGTTTTCTGAGCGGTCGTGCCAAGGCATCAGCCCAGCTGGAAACGATCAAAACGAAA   180
ptsI_LF82        GAACGTTTTCTGAGCGGTCGTGCCAAGGCATCAGCCCAGCTGGAAACGATCAAAACGAAA   180
                 ************************************************************

ptsI_MG1655      GCTGGTGAAACGTTCGGTGAAGAAAAAGAAGCCATCTTTGAAGGGCATATTATGCTGCTC   240
ptsI_NRG857c     GCTGGTGAAACGTTCGGTGAAGAAAAAGAAGCCATCTTTGAAGGGCATATTATGCTGCTC   240
ptsI_LF82        GCTGGTGAAACGTTCGGTGAAGAAAAAGAAGCCATCTTTGAAGGGCATATTATGCTGCTC   240
                 ************************************************************

ptsI_MG1655      GAAGATGAGGAGCTGGAGCAGGAAATCATAGCCCTGATTAAAGATAAGCACATGACAGCT   300
ptsI_NRG857c     GAAGATGAGGAGCTGGAGCAGGAAATCATAGCCCTGATTAAAGATAAGCACATGACAGCT   300
ptsI_LF82        GAAGATGAGGAGCTGGAGCAGGAAATCATAGCCCTGATTAAAGATAAGCACATGACAGCT   300
                 ************************************************************

ptsI_MG1655      GACGCAGCTGCTCATGAAGTTATCGAAGGTCAGGCTTCTGCCCTGGAAGAGCTGGATGAT   360
ptsI_NRG857c     GACGCAGCTGCTCATGAAGTTATCGAAGGTCAGGCTTCTGCCCTGGAAGAGCTGGATGAT   360
ptsI_LF82        GACGCAGCTGCTCATGAAGTTATCGAAGGTCAGGCTTCTGCCCTGGAAGAGCTGGATGAT   360
                 ************************************************************

ptsI_MG1655      GAATACCTGAAAGAACGTGCGGCTGACGTACGTGATATCGGTAAGCGCCTGCTGCGCAAC   420
ptsI_NRG857c     GAATACCTGAAAGAACGTGCGGCTGACGTACGTGATATCGGTAAGCGCCTGCTGCGCAAC   420
ptsI_LF82        GAATACCTGAAAGAACGTGCGGCTGACGTACGTGATATCGGTAAGCGCCTGCTGCGCAAC   420
                 ************************************************************

ptsI_MG1655      ATCCTGGGCCTGAAGATTATCGACCTGAGCGCCATTCAGGATGAAGTCATTCTGGTTGCC   480
ptsI_NRG857c     ATCCTGGGCCTGAAGATTATCGACCTGAGCGCCATTCAGGATGAAGTCATTCTGGTTGCC   480
ptsI_LF82        ATCCTGGGCCTGAAGATTATCGACCTGAGCGCCATTCAGGATGAAGTCATTCTGGTTGCC   480
                 ************************************************************

ptsI_MG1655      GCTGACCTGACGCCGTCCGAAACCGCACAGCTGAACCTGAAGAAGGTGCTGGGTTTCATC   540
ptsI_NRG857c     GCTGACCTGACGCCGTCCGAAACCGCACAGCTGAACCTGAAGAAGGTGCTGGGTTTCATC   540
ptsI_LF82        GCTGACCTGACGCCGTCCGAAACCGCACAGCTGAACCTGAAGAAGGTGCTGGGTTTCATC   540
                 ************************************************************

ptsI_MG1655      ACCGACGCGGGTGGCCGTACTTCCCACACCTCTATCATGGCGCGTTCTCTGGAACTACCT   600
ptsI_NRG857c     ACCGACGCGGGTGGCCGTACTTCCCACACCTCTATCATGGCGCGTTCTCTGGAACTGCCT   600
ptsI_LF82        ACCGACGCGGGTGGCCGTACTTCCCACACCTCTATCATGGCGCGTTCTCTGGAACTGCCT   600
                 ******************************************************** ***

ptsI_MG1655      GCTATCGTGGGTACCGGTAGCGTCACCTCTCAGGTGAAAAATGACGACTATCTGATTCTG   660
ptsI_NRG857c     GCTATCGTGGGTACCGGTAGCGTCACCTCTCAGGTGAAAAATGACGACTATCTGATTCTG   660
ptsI_LF82        GCTATCGTGGGTACCGGTAGCGTCACCTCTCAGGTGAAAAATGACGACTATCTGATTCTG   660
                 ************************************************************

ptsI_MG1655      GATGCCGTAAATAATCAGGTTTACGTCAATCCAACCAACGAAGTTATTGATAAAATGCGC   720
ptsI_NRG857c     GATGCCGTAAATAATCAGGTTTACGTCAATCCAACCAACGAAGTTATTGATAAAATGCGC   720
ptsI_LF82        GATGCCGTAAATAATCAGGTTTACGTCAATCCAACCAACGAAGTTATTGATAAAATGCGC   720
                 ************************************************************

ptsI_MG1655      GCTGTTCAGGAGCAAGTGGCTTCTGAAAAAGCAGAGCTTGCTAAACTGAAAGATCTGCCA   780
ptsI_NRG857c     GCTGTTCAGGAGCAAGTGGCTTCTGAAAAAGCAGAGCTTGCTAAACTGAAAGATCTGCCA   780
ptsI_LF82        GCTGTTCAGGAGCAAGTGGCTTCTGAAAAAGCAGAGCTTGCTAAACTGAAAGATCTGCCA   780
                 ************************************************************

ptsI_MG1655      GCTATTACGCTGGACGGTCACCAGGTAGAAGTATGCGCTAACATTGGTACGGTTCGTGAC   840
ptsI_NRG857c     GCTATTACGCTGGACGGTCACCAGGTAGAAGTGTGCGCTAACATTGGTACGGTTCGTGAC   840
ptsI_LF82        GCTATTACGCTGGACGGTCACCAGGTAGAAGTGTGCGCTAACATTGGTACGGTTCGTGAC   840
                 ******************************** ***************************

ptsI_MG1655      GTTGAAGGTGCAGAGCGTAACGGCGCTGAAGGCGTTGGTCTGTATCGTACTGAGTTCCTG   900
ptsI_NRG857c     GTTGAAGGTGCAGAGCGTAACGGCGCTGAAGGCGTTGGTCTGTATCGTACTGAGTTCCTG   900
ptsI_LF82        GTTGAAGGTGCAGAGCGTAACGGCGCTGAAGGCGTTGGTCTGTATCGTACTGAGTTCCTG   900
                 ************************************************************
```

```
ptsI_MG1655        TTCATGGACCGCGACGCACTGCCCACTGAAGAAGAACAGTTTGCTGCTTACAAAGCAGTG   960
ptsI_NRG857c       TTCATGGACCGCGACGCGCTGCCCACTGAAGAAGAACAGTTTGCTGCTTACAAAGCAGTG   960
ptsI_LF82          TTCATGGACCGCGACGCGCTGCCCACTGAAGAAGAACAGTTTGCTGCTTACAAAGCAGTG   960
                   ***************** ******************************************

ptsI_MG1655        GCTGAAGCGTGTGGCTCGCAAGCGGTTATCGTTCGTACCATGGACATCGGCGGCGACAAA   1020
ptsI_NRG857c       GCTGAAGCGTGTGGCTCTCAGGCGGTTATCGTTCGTACCATGGACATCGGCGGCGACAAA   1020
ptsI_LF82          GCTGAAGCGTGTGGCTCTCAGGCGGTTATCGTTCGTACCATGGACATCGGCGGCGACAAA   1020
                   ***************** ** ***************************************

ptsI_MG1655        GAGCTGCCATACATGAACTTCCCGAAAGAAGAGAACCCGTTCCTCGGCTGGCGCGCTATC   1080
ptsI_NRG857c       GAGCTGCCATACATGAACTTCCCGAAAGAAGAGAACCCGTTCCTCGGCTGGCGCGCTATC   1080
ptsI_LF82          GAGCTGCCATACATGAACTTCCCGAAAGAAGAGAACCCGTTCCTCGGCTGGCGCGCTATC   1080
                   ************************************************************

ptsI_MG1655        CGTATCGCGATGGATCGTAGAGAGATCCTGCGCGATCAGCTCCGCGCTATCCTGCGTGCC   1140
ptsI_NRG857c       CGTATCGCGATGGATCGTAAAGAGATCCTGCGCGATCAGCTCCGCGCTATCCTGCGTGCC   1140
ptsI_LF82          CGTATCGCGATGGATCGTAAAGAGATCCTGCGCGATCAGCTCCGCGCTATCCTGCGTGCC   1140
                   ******************* ****************************************

ptsI_MG1655        TCGGCTTTCGGTAAATTGCGCATTATGTTCCCGATGATCATCTCTGTTGAAGAAGTGCGT   1200
ptsI_NRG857c       TCTGCTTTCGGTAAATTGCGCATCATGTTCCCGATGATCATCTCTGTTGAAGAAGTGCGT   1200
ptsI_LF82          TCTGCTTTCGGTAAATTGCGCATCATGTTCCCGATGATCATCTCTGTTGAAGAAGTGCGT   1200
                   ** ******************** ************************************

ptsI_MG1655        GCACTGCGCAAAGAGATCGAAATCTACAAACAGGAACTGCGCGACGAAGGTAAAGCGTTT   1260
ptsI_NRG857c       GCACTGCGCAAAGAGATCGAAATCTACAAACAGGAACTGCGCGACGAAGGTAAAGCGTTT   1260
ptsI_LF82          GCACTGCGCAAAGAGATCGAAATCTACAAACAGGAACTGCGCGACGAAGGTAAAGCGTTT   1260
                   ************************************************************

ptsI_MG1655        GACGAGTCAATTGAAATCGGCGTAATGGTGGAAACACCGGCTGCCGCAACAATTGCACGT   1320
ptsI_NRG857c       GACGAGTCAATTGAAATCGGCGTAATGGTGGAAACACCGGCTGCCGCAACAATTGCACGT   1320
ptsI_LF82          GACGAGTCAATTGAAATCGGCGTAATGGTGGAAACACCGGCTGCCGCAACAATTGCACGT   1320
                   ************************************************************

ptsI_MG1655        CATTTAGCCAAAGAAGTTGATTTCTTTAGTATCGGCACCAATGATTTAACGCAGTACACT   1380
ptsI_NRG857c       CATTTAGCCAAAGAAGTTGATTTCTTTAGTATCGGCACCAATGATTTAACGCAGTACACT   1380
ptsI_LF82          CATTTAGCCAAAGAAGTTGATTTCTTTAGTATCGGCACCAATGATTTAACGCAGTACACT   1380
                   ************************************************************

ptsI_MG1655        CTGGCAGTTGACCGTGGTAATGATATGATTTCACACCTTTACCAGCCAATGTCACCGTCC   1440
ptsI_NRG857c       CTGGCAGTTGACCGTGGTAATGATATGATTTCACACCTTTACCAGCCAATGTCACCGTCC   1440
ptsI_LF82          CTGGCAGTTGACCGTGGTAATGATATGATTTCACACCTTTACCAGCCAATGTCACCGTCC   1440
                   ************************************************************

ptsI_MG1655        GTGCTGAACTTGATCAAGCAAGTTATTGATGCTTCTCATGCTGAAGGCAAATGGACTGGC   1500
ptsI_NRG857c       GTGCTGAACTTGATCAAGCAAGTTATTGATGCTTCTCATGCTGAAGGCAAATGGACTGGC   1500
ptsI_LF82          GTGCTGAACTTGATCAAGCAAGTTATTGATGCTTCTCATGCTGAAGGCAAATGGACTGGC   1500
                   ************************************************************

ptsI_MG1655        ATGTGTGGTGAGCTTGCTGGCGATGAACGTGCTACACTTCTGTTGCTGGGGATGGGTCTG   1560
ptsI_NRG857c       ATGTGTGGTGAGCTTGCTGGCGATGAACGTGCTACACTTCTGTTGCTGGGGATGGGTCTG   1560
ptsI_LF82          ATGTGTGGTGAGCTTGCTGGCGATGAACGTGCTACACTTCTGTTGCTGGGGATGGGTCTG   1560
                   ************************************************************

ptsI_MG1655        GACGAATTCTCTATGAGCGCCATTTCTATCCCGCGCATTAAGAAGATTATCCGTAACACG   1620
ptsI_NRG857c       GACGAATTCTCTATGAGCGCCATTTCTATCCCGCGCATTAAGAAGATTATCCGTAACACG   1620
ptsI_LF82          GACGAATTCTCTATGAGCGCCATTTCTATCCCGCGCATTAAGAAGATTATCCGTAACACG   1620
                   ************************************************************

ptsI_MG1655        AACTTCGAAGATGCGAAGGTGTTAGCAGAGCAGGCTCTTGCTCAACCGACAACGGACGAG   1680
ptsI_NRG857c       AACTTCGAAGATGCGAAGGTGTTAGCAGAGCAGGCTCTTGCTCAACCGACAACGGACGAG   1680
ptsI_LF82          AACTTCGAAGATGCGAAGGTGTTAGCAGAGCAGGCTCTTGCTCAACCGACAACGGACGAG   1680
                   ************************************************************


ptsI_MG1655        TTAATGACGCTGGTTAACAAGTTCATTGAAGAAAAAACAATCTGCTAA 1728
ptsI_NRG857c       TTAATGACGCTGGTTAACAAGTTCATTGAAGAAAAAACAATCTGCTAA 1728
ptsI_LF82          TTAATGACGCTGGTTAACAAGTTCATTGAAGAAAAAACAATCTGCTAA 1728
                   ************************************************
```

SUPPLEMENTAL MATERIALS AND METHODS

Bacterial growth curves in MOPS-minimal medium

Bacterial strains were inoculated from a saturated culture into MOPS-based medium supplemented with 0.4% glucose prepared as described in [~~1~~18]. Growth curves were done in 96-well plates incubated in a TECAN spectrophotometer (Infinity 2000, TECAN) and started at OD_{600nm} 0.03 (to resemble killing curves conditions). Plates were incubated at 37°C with shaking, and density was measured each 10 min for a total period of 24 h. Data from triplicate cultures were analyzed in GraphPad Prism 10 Version 10.3.0.

Time-killing curves in MOPS-based medium

E. coli NRG857c overnight cultures were inoculated from frozen glycerol stocks into 2 ml of MOPS-based medium and grown overnight (18–20 hours) at 37°C with shaking at 170 rpm. Fresh MOPS-based medium was inoculated at a starting OD_{600nm} of 0.03, and growth was continued until reaching the early exponential growth phase (OD_{600nm} 0.3-0.4) with good aeration. Ciprofloxacin was added to 0.15, 0.30 or 0.60 μg/mL to each culture and grown up to 5 h at 37°C with aeration. Samples were taken at several time points after antibiotic treatment, serially diluted in PBS and plated on LB-agar without antibiotic. After incubation at 37°C, CFU/ml were determined, and the survival ratio was graphed as a function of time.

PCR amplification and sequencing of *hipA* alleles from bacteria recovered after antibiotic treatment

hipA genes were PCR amplified from selected NRG857c bacterial colonies recovered 3-hour post-antibiotic treatment. PCR amplifications were performed using MangoMix (Bioline) according to the manufacturer's instructions with minor modifications. Briefly, each reaction mixture contained 1x MangoMix, 0.5 μM of each forward (5'-AGCAGGCGACAATTTCCAAT-3') and reverse (5'-AAGGGAGAATCCAGTCGTTG-3') primer, and 1 μL of template (bacterial lysates). Amplifications were carried out under the following cycling conditions: initial denaturation at 95 °C for 5 minutes, followed by 30 cycles of denaturation at 95 °C for 30 seconds, annealing at 55 °C for 30 seconds, and extension at 72 °C for 30 seconds. PCR products were verified by electrophoresis on a 1% agarose gel stained with GelRed (Biotium) and visualized under UV illumination. Purified amplicons (FavorPrep GEL/PCR Purification Kit, Favorgen Biotech Corp) were submitted for Sanger sequencing (Macrogen, Chile), and sequencing results aligned to the reference sequence using the SnapGene software version 8.0.2.

Bioinformatic analysis

Multiple sequence alignments were done using the Clustal Omega (1.2.4) program available at EMBL-EBI web site [~~2~~38].

REFERENCES

18. Goormaghtigh F, and Van Melderen L (**2016**). Optimized Method for Measuring Persistence in Escherichia coli with Improved Reproducibility. In: Michiels J, Fauvart M, editors Bacterial Persistence. **Springer New York, New York, NY**; pp 43–52. doi: 10.1007/978-1-4939-2854-5_4

38. Madeira F, Madhusoodanan N, Lee J, Eusebi A, Niewielska A, Tivey ARN, Lopez R, and Butcher S (**2024**). The EMBL-EBI Job Dispatcher sequence analysis tools framework in 2024. **Nucleic Acids Research** 52(W1): W521–W525. doi: 10.1093/nar/gkae241
